# Complement in acute kidney injury: a convergent pathogenic pathway in multifactorial renal damage

**DOI:** 10.3389/fimmu.2026.1840335

**Published:** 2026-05-22

**Authors:** Dan Yi, Rong Yang, Yue Guo, Hua Zhou, Junjun Luan

**Affiliations:** 1 Department of Nephrology, Shengjing Hospital of China Medical University, Shenyang, China; 2 Key Laboratory of Environmental Stress and Chronic Disease Control and Prevention, Ministry of Education (China Medical University), Shenyang, China

**Keywords:** acute kidney injury, C3/C5 axis, complement system, ischemia-reperfusion injury, sepsis-associated AKI, tubular injury

## Abstract

Acute kidney injury (AKI) is a common clinical syndrome characterized by substantial etiologic heterogeneity, whose pathophysiology encompasses inflammatory amplification, microcirculatory dysfunction, tubular injury, and maladaptive repair. Evidence from preclinical models and clinical samples indicates that dysregulated complement activation represents a potential convergent mechanism of injury in multiple forms of AKI, although its relative contribution varies across etiologies. Experimental models support mechanistic and pathogenic roles for complement activation, whereas clinical evidence in many AKI settings remains largely associative, suggesting a contributory rather than universally causal role. Diverse insults, including ischemia-reperfusion injury, nephrotoxins, and sepsis, can initiate complement activation, which converges on C3 and C5, fueling inflammatory escalation and tissue injury. In addition to cytotoxicity mediated by the terminal pathway, the complement system participates in the initiation, progression, and outcome of injury by orchestrating endothelial activation, leukocyte recruitment, tubular epithelial stress, and innate immune amplification. Concurrently, dysregulation of complement regulatory proteins, persistent activation of the C5a/C5aR axis, and crosstalk with Toll-like receptor signaling and neutrophil extracellular trap (NET) formation further amplify the damaging effects, thereby exacerbating AKI progression. Moreover, complement activation exhibits pronounced spatiotemporal heterogeneity, with its pathogenic effects shaped by the renal compartment, injury stage, and persistence of activation. This review summarizes the dynamic regulatory network of complement activation and its pathogenic mechanisms across diverse AKI etiologies, providing an integrative framework for biomarker-guided stratification and precision therapy in AKI.

## 1 Introduction

Acute kidney injury (AKI) is a common clinical syndrome in hospitalized patients, particularly in critical care settings (1). It is associated with high morbidity and mortality, contributing to more than 1.5 million deaths worldwide annually (2, 3). Predisposing conditions include hypovolemia, crush injury, and pre-existing chronic diseases (4). Clinically, AKI is defined by increased serum creatinine and/or a decline in urine output, reflecting an acute decline in glomerular filtration rate (5). Histopathologically, AKI is characterized predominantly by acute tubular injury, manifesting as tubular lumen dilatation and loss of the brush border in the proximal tubules, which may further trigger innate immune activation (6). Despite substantial etiologic heterogeneity, distinct injurious stimuli converge on shared downstream pathogenic pathways, including inflammatory amplification, microcirculatory dysfunction, tubular epithelial injury, and maladaptive repair (7).

In the pathogenesis of AKI, the complement system has emerged as an important pathogenic contributor rather than a mere secondary epiphenomenon (8), although the strength of evidence varies across models, clinical contexts, and etiologies, ranging from causal involvement to associative or contributory roles. As a key effector arm of innate immunity, complement can be activated by multiple AKI-relevant insults and propagate renal injury through convergent effector cascades centered on C3 and C5 (9). Furthermore, dysregulated complement activation exacerbates oxidative stress and microcirculatory dysfunction, fueling a feed-forward cycle of tissue injury and inflammation (10). Collectively, these observations suggest that complement activation may represent a potential convergent mechanism of injury across multiple etiologies of AKI, although its relative importance likely varies with the underlying cause and stage of injury.

In this review, we synthesize current evidence on the role of complement in AKI initiation and progression, while distinguishing its mechanistic, causal, and associative implications according to evidence source and strength. We focus on shared and etiology-specific mechanisms across major AKI contexts, including ischemia-reperfusion injury, sepsis-associated AKI, and nephrotoxic AKI, and further evaluate the potential of complement-targeted strategies for biomarker-guided stratification and precision therapy.

## 2 The renal complement system: activation pathways, local production, and regulatory networks

### 2.1 Overview of complement activation pathways

The complement system comprises over 30 soluble and membrane-bound proteins (11) and functions as a frontline sensor of tissue injury, with essential roles in host defense and tissue homeostasis (12). However, dysregulated complement activation can also drive tissue injury and maladaptive inflammation (13). Complement activation occurs through three major pathways: the classical, lectin, and alternative pathways, all of which converge on the central components C3 and C5 and ultimately produce bioactive effector fragments (14) that orchestrate inflammatory, vasoactive, and immunometabolic responses (15).

The classical pathway is typically triggered by immune complexes, apoptotic debris, or target antigens recognized by natural antibodies (16). These stimuli activate the C1 complex, thereby triggering cleavage of C4 and C2 and assembly of the classical C3 convertase (C4bC2b), ultimately culminating in membrane attack complex (MAC) assembly and target cell lysis (17, 18).

The lectin pathway is activated by mannose-binding lectin (MBL), collectins, or ficolins upon recognition of glycan motifs on microbial or stressed host cell surfaces (19). This pathway relies on MASP-1 and MASP-2 for C4bC2b assembly (20) and also promotes amplification of the alternative pathway (21). Notably, mannose-binding lectin-associated serine proteases (MASPs) and MBL-associated protein 1 can interact with collectin-11. Under conditions of cellular stress, TEC-derived collectin-11 recognizes *Fut2*-dependent fucosylated ligands and triggers lectin pathway activation (22, 23).

**Figure 1:**
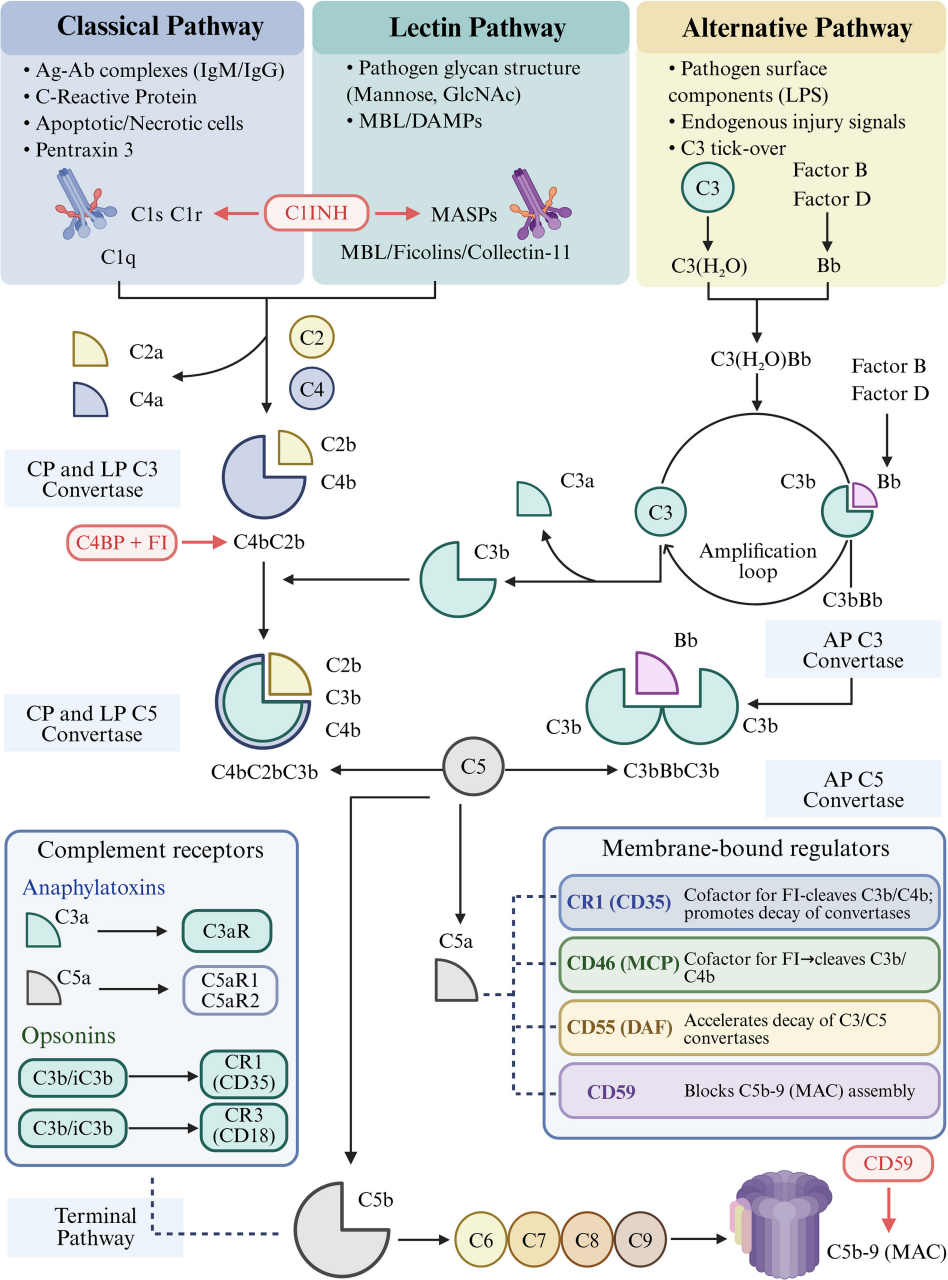
Schematic illustration of complement activation pathways, regulatory networks, and effector signaling. The complement system is activated through three pathways: the classical pathway (left) is triggered by antigen-antibody complex binding to C1q. The lectin pathway (middle) activates MASPs after recognition of pathogen surface molecules by MBL/ficolins, and the two pathways activate C4/C2 to form C4bC2b. The alternative pathway (right) is initiated by the spontaneous hydrolysis of C3, and the combination of C3b with factor B/D to form C3bBb. The classical and lectin pathways generate C4bC2b, whereas the alternative pathway generates C3bBb. These pathways ultimately generate C5 convertases, such as C4bC2bC3b and C3bBbC3b, which cleave C5 and initiate MAC assembly. Soluble and membrane-bound regulators, including FH, FI, C1INH, C4BP, CR1/CD35, CD46/MCP, CD55/DAF, and CD59, restrain complement activation at different levels, whereas complement receptors such as C3aR, C5aR1/C5aR2, CR1, and CR3 mediate downstream inflammatory and cellular responses. This Figure is created with https://www.BioRender.com.

The alternative pathway is initiated through spontaneous C3 tick-over, followed by factor B binding and C3 convertase C3bBb assembly (24). In addition to promoting MAC formation and generation of the anaphylatoxins C3a and C5a (25), it functions both as a constitutive surveillance mechanism on renal endothelial and epithelial surfaces and as the principal amplification loop for the classical and lectin pathways. As such, it contributes substantially to C3 consumption and downstream tissue injury during acute renal stress (26) (**Figure 1**).

### 2.2 Complement regulatory networks and effector signaling in AKI

Complement activation is controlled by soluble regulators, including factor H (FH), factor I (FI), C1 inhibitor (C1INH), C4-binding protein (C4BP), and properdin, as well as membrane-bound regulators, including CR1/CD35, CD46/MCP, CD55/DAF, and CD59 (27, 28). C1INH restricts the initiation of the classical and lectin pathways by inactivating C1r/C1s and MASPs, whereas C4BP and FI inhibit the formation of C4b-dependent convertases (29). FH acts as an FI cofactor to promote C3b cleavage and C3 convertase decay, thereby suppressing alternative pathway amplification and downstream C5b-9 formation (30, 31). FH can also bind to uromodulin and may participate in the transition from AKI to chronic kidney disease (CKD) (32). Under pathological conditions such as sepsis, FH and FH-related proteins (FHRs) may further influence the surface localization of complement activation (33). In contrast, properdin is the only positive regulator of the alternative pathway. It can bind to the surface of renal tubular epithelial cells (TECs) and promote local alternative pathway activation (34). By stabilizing C3/C5 convertases and prolonging the half-life of C3bBb, properdin amplifies complement responses initiated by the classical and lectin pathways (35, 36). On the cell surface, CR1/CD35, CD46/MCP, and CD55/DAF limit complement activation by accelerating convertase decay or acting as cofactors for FI, whereas CD59 prevents MAC assembly (27). Beyond soluble effector fragments, complement also signals through receptors such as C3aR, C5aR1/C5aR2, CR1, and CR3, linking complement activation to leukocyte recruitment, phagocytosis, endothelial activation, and renal tubular inflammation (37).

### 2.3 Systemic complement and intrarenal sources of complement

Although the liver is the principal source of circulating complement, the kidney also serves as an extrahepatic source of complement components. Renal tubules represent a major site of intrarenal complement expression under both physiological and pathological conditions (38). Intrarenal complement regulation is highly refined yet inherently fragile. During AKI, renal complement accumulation reflects not only plasma-derived influx but also enhanced intrarenal synthesis, particularly through increased tubular expression of C3, C4, and factor B (5). Mesangial cells and macrophages may further reinforce local complement amplification (39). Moreover, disruption of tubular epithelial polarity can expose previously protected cell surfaces to alternative pathway attack, thereby facilitating uncontrolled complement activation within the injured microenvironment (40). As a result, intrarenal complement production generates high local concentrations of active fragments and convertases, amplifying inflammation and tissue injury beyond that attributable to circulating complement alone (41).

### 2.4 Compartment-specific complement regulation in the kidney

To prevent host tissue injury, the kidney relies on a compartmentalized network of soluble and membrane-bound complement regulators. Given its constant exposure to circulating complement, the vascular compartment relies primarily on factor H recruitment to endothelial heparan sulfate, together with membrane-bound regulators, to limit spontaneous alternative pathway activation (42). The glomerulus is relatively enriched in CR1, CD46, CD55, and CD59, which help preserve filtration barrier integrity and limit C3/C5 convertase activity as well as C5b-9 formation (43). By contrast, the tubulointerstitial compartment displays comparatively limited basal regulatory capacity. In tubular epithelial cells, complement regulation is highly polarized: in humans, it relies predominantly on basolateral CD46 (44), whereas in rodents, the broadly expressed complement receptor 1-related protein y (Crry) serves as the dominant functional regulator (45). During AKI, this compartmentalized regulatory architecture is highly susceptible to disruption by epithelial injury and loss of polarity, thereby promoting local complement amplification and exacerbating tissue injury (5).

## 3 Role of complement in AKI across diverse etiologies

AKI arises from diverse etiologies with distinct pathophysiological features. Renal ischemia, nephrotoxins, sepsis, hemolysis/rhabdomyolysis, and virus-associated AKI, including SARS-CoV-2 infection, are major contexts in which complement activation has been implicated in AKI initiation and progression. However, the evidence varies by etiology: experimental ischemia-reperfusion models provide strong mechanistic support for a pathogenic role, whereas clinical evidence in sepsis-associated and virus-associated AKI, particularly SARS-CoV-2-associated AKI, mainly derives from biomarkers, tissue deposition, or cohort observations, supporting associations with disease severity and injury progression (**Table 1**).

**Table 1:** The influence of complement activation on AKI induced by various triggers.

Inducing factor	Main pathways	Key signaling pathways	Pathogenic mechanism	References
Ischemia-reperfusion	CP/AP/LP	C5a/C5aR TLR4/NF-κB PI3K/Akt	Endothelial damage/DAMPs→ Complement response → Tubular injury/AKI risk↑	(46–49)
Cisplatin	AP	C5a/C5aR	Complement activation→ Tubular toxicity→ AKI severity↑	(50–53)
Contrast agents	LP	HIF-1α/NOX2	MBL-glycocalyx interaction→ Renal medullary hypoperfusion → AKI risk↑	(54, 55)
Animal toxin	CP/AP/LP	TLR4/NF-κB TNF-α	Proteases cleave complement→ Complement activation → AKI risk↑	(56)
Sepsis	CP/AP/LP	TLR4/NF-κB TNF-α	PAMPs/DAMPs→ C3a/C5a ↑/Inflammatory response → Complement activation→ Renal microvascular collapse/AKI severity↑	(57, 58)
Intravascular hemolysis	CP/AP	TLR4/NF-κB	Heme↑/Iron-mediated oxidative stress→ Complement activation→ AKI severity↑	(59)
Rhabdomyolysis	CP/AP	Nrf2/HO-1 TLR4/NF-κB	Heme↑/Iron-mediated oxidative stress→ Complement activation→ AKI severity↑	(60)
Virus-associated AKI	AP/LP	C5a/C5aR TLR4/NF-κB	Viral complement activation → inflammation/immune-complex injury → AKI severity↑	(61–64)

The up arrow (↑) indicates an increase, and the arrow (→) indicates the direction of progression. CP, classical pathway; AP, alternative pathway; LP, lectin pathway; PI3K, phosphoinositide 3-kinase; HIF-1α, hypoxia-inducible factor 1α; NOX2, NADPH oxidase 2; TNF-α, tumor necrosis factor-α; Nrf2, nuclear factor erythroid 2-related factor 2; HO-1, heme oxygenase-1.

### 3.1 Ischemia-reperfusion-induced AKI

Ischemia-reperfusion (IR) injury is a classic cause of AKI, characterized by acute renal parenchymal injury driven by the combined effects of post-reperfusion oxidative stress, endothelial dysfunction, and sterile inflammation (65). In ischemia-reperfusion-induced AKI (IR-AKI), experimental studies involving genetic deficiency, complement blockade, and complement regulator modulation support complement activation as an important injury amplifier (66), whereas direct clinical evidence remains limited.

#### 3.1.1 Complement-mediated endothelial dysfunction and microvascular injury

During the early phase of IR, complement activation primarily targets the renal microvasculature and endothelial compartment. The classical pathway can be activated on injured endothelial surfaces and contributes to endothelial-to-mesenchymal transition (67). In this context, C3a and C5a drive endothelial phenotypic remodeling through signaling pathways such as Akt, increase vascular permeability, aggravate local hypoxia, and thereby impair tubular function (68, 69). Furthermore, C3a/C5a promote the expression of pro-inflammatory mediators such as IL-6, thereby enhancing leukocyte recruitment and endothelial activation and exacerbating microcirculatory dysfunction (70). Notably, ceramide transporters have been shown to bind C1q, suggesting that lipid stress may also contribute to early classical pathway activation during IR injury (71).

#### 3.1.2 Complement-mediated tubular inflammation and cell death

As injury progresses, the dominant site of complement activation shifts to the tubular compartment. Following ischemia, the lectin pathway-associated molecule Collectin-11 (CL-11) is upregulated on the basolateral surface of the proximal tubule and colocalizes with complement deposits, suggesting a central role in local complement recognition within the tubular compartment (72). Conversely, Tamm-Horsfall protein (THP) may confer endogenous protection against complement-mediated tubular injury through multiple mechanisms. THP can interfere with the fucoidan-dependent binding of CL-11, thereby limiting lectin pathway activation (73). It may also interact with C1q and inhibit classical pathway activation (74). In addition, THP serves as a cofactor for factor I-mediated C3b cleavage (75).

In parallel, increased local ammonia production following IR (76), properdin deposition on the tubular surface (34), and factor B/factor D-dependent stabilization of C3bBb sustain C3 cleavage and amplify local complement activation (77, 78). As the only known positive regulator of the alternative pathway, properdin not only binds to the apical surface of TECs and facilitates local complement activation, but also further intensifies inflammatory responses by modulating the phagocytosis of damaged cells (78, 79). At the downstream effector stage, C5a acts as a potent pro-inflammatory mediator through C5aR1 expressed on inflammatory cells and tubular epithelial cells, thereby promoting inflammatory cell recruitment and aggravating tubular inflammation (46, 80). The terminal complement complex C5b-9 further exacerbates TEC injury and necrotic cell death. Moreover, in mice with impaired C5b-9 production, IR-induced kidney injury was significantly reduced (81).

In mouse IR models and experimental hypoxia systems, ischemia or chemical hypoxia can reduce Crry expression on TEC surfaces (34), thereby weakening C3b/C4b inactivation and convertase regulation and promoting local complement activation and interstitial inflammation (34, 82, 83). As Crry is the dominant membrane complement regulator in rodents, these findings should be interpreted as murine/preclinical evidence rather than direct human data. In these models, Crry and factor H synergistically restrain basolateral complement activation in TECs. Conversely, Crry insufficiency or impaired factor H binding aggravates ischemic injury, whereas factor H supplementation is protective (84, 85).

#### 3.1.3 Bidirectional crosstalk between complement and sterile inflammatory signaling

**Figure 2:**
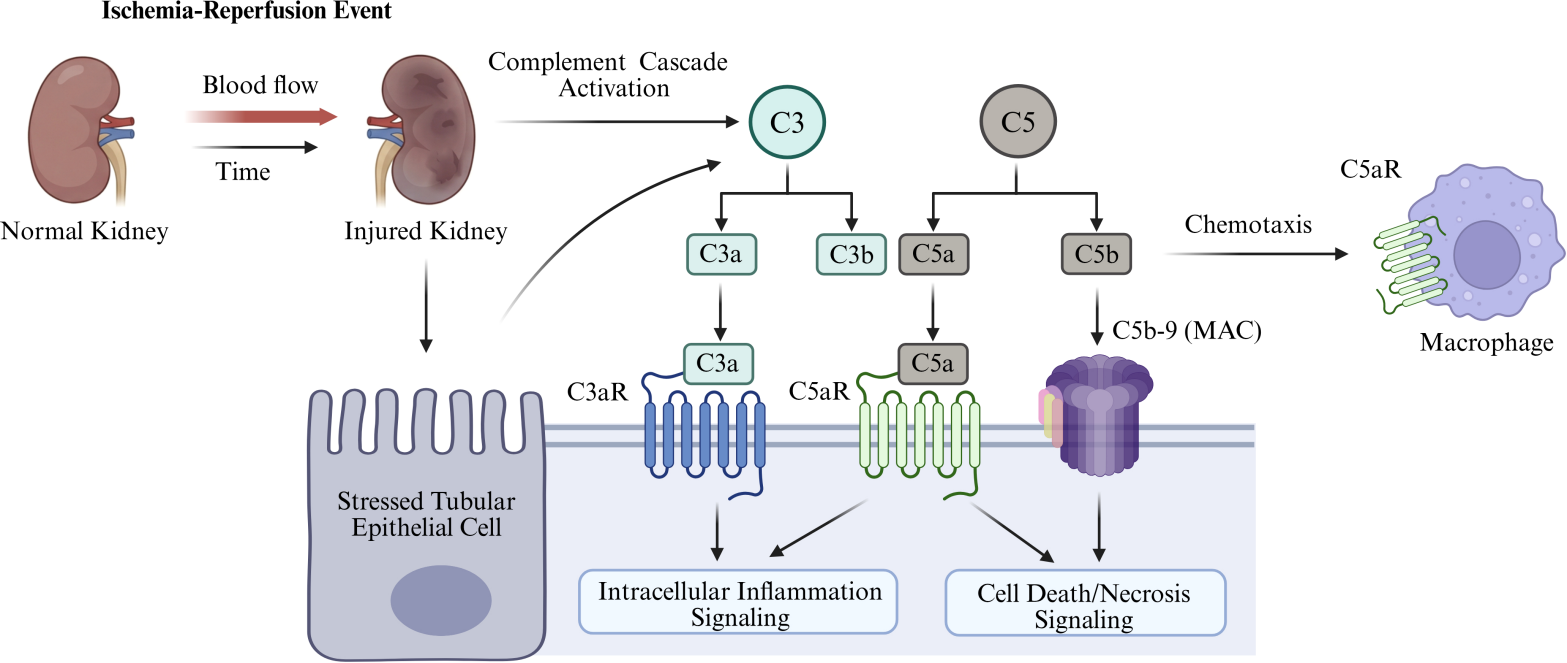
Mechanism of ischemia-reperfusion-induced complement activation leading to AKI. Following ischemia–reperfusion injury, complement cascade activation in the kidney generates the effector fragments C3a, C5a, and C5b-9 (MAC). C3a and C5a signal through C3aR and C5aR on stressed tubular epithelial cells to promote intracellular inflammatory responses, while C5a also recruits macrophages via chemotaxis. In parallel, C5b-9 directly damages tubular epithelial cells and triggers cell death and necrosis signaling, thereby aggravating acute kidney injury. This Figure is created with https://www.BioRender.com.

In IR-AKI, complement activation is closely integrated with sterile inflammatory signaling rather than acting as an isolated cascade. Reperfusion-induced reactive oxygen species (ROS) and pro-inflammatory cytokine release can potentiate complement activation (86), while uncontrolled complement activation further weakens local inhibitory mechanisms, including factor H-dependent regulation, thereby reinforcing tissue inflammation (87). Experimental studies also link C3a signaling (47) and alternative pathway activation to neutrophil extracellular trap (NET) formation, tubular necrosis (88), and post-injury inflammatory remodeling (89). These findings support a feed-forward interaction between complement and sterile inflammation in IR-AKI, but this conclusion is derived primarily from experimental models (**Figure 2**).

### 3.2 Nephrotoxin-induced AKI

Nephrotoxin-induced AKI is commonly triggered by cisplatin (90), iodinated contrast agents (91), and biological toxins such as snake venom (92), bee venom (93), and fish bile toxin (94). Most cases are characterized by secondary tubular injury with predominant involvement of the proximal tubules in the cortex and outer medulla, and may be accompanied by complement activation that amplifies necrosis, apoptosis, inflammation, and later fibrotic remodeling (95, 96). Among these insults, cisplatin enters proximal tubular cells via organic cation transporter 2 and organic anion transporters, causing dose-dependent nephrotoxicity (97, 98), whereas contrast-induced AKI (CI-AKI) is mainly associated with renal medullary hypoxia and direct cytotoxicity (91). Current evidence suggests that complement activation may contribute to tubular injury, microvascular dysfunction, and impaired tissue repair in nephrotoxin-induced AKI, although the evidence base differs among insults: cisplatin-related mechanisms are supported largely by experimental studies, whereas CI-AKI studies more often link complement biomarkers with renal injury severity in clinical settings (99, 100). In particular, beyond direct nephrotoxicity (101), wasp venom can induce intravascular hemolysis and rhabdomyolysis, with the resulting release of free hemoglobin and myoglobin exacerbating pigment-mediated tubular injury and potentially amplifying complement-mediated renal damage (102). Proteomic and metabolomic evidence further links this secondary injury to venom-induced membrane glycerophospholipid disruption, whereas phospholipase inhibition alleviates hemolysis, rhabdomyolysis, renal inflammation, and kidney injury (103, 104).

#### 3.2.1 Complement activation pathways in drug-induced AKI: cisplatin and contrast agents

Both cisplatin- and iodinated contrast agent-induced AKI have been implicated in dysregulated complement activation, although the supporting evidence differs between these settings. In cisplatin-induced AKI, current evidence does not establish a single dominant activation pathway. Experimental studies have reported increased C1s/IgG, MBL1/C4 fragments (105), as well as significant upregulation of transcripts encoding C1q, C2, C3, and C4 in mouse kidneys, accompanied by increased C3a and C5a generation (106). These findings suggest activation of the C3/C5 effector axis, which may contribute to tubular inflammation and cellular injury (50). Cisplatin-induced mitochondrial damage and oxidative stress may further promote release of damage-associated molecular patterns (DAMPs) (107), while CL-11 can activate the lectin pathway through interactions with MASPs (108). Cisplatin administration also increases renal caspase expression (51), while N-acetylcysteine may attenuate the inflammatory response by inhibiting the C5a/C5aR pathway (109). Complement-modulating interventions, such as caloric restriction, can attenuate C3/C4 cleavage and C5a generation, lower levels of inflammatory cytokines, maintain renal tubular membrane integrity, and mitigate complement-mediated injury (90).

In CI-AKI, elevated C1q levels should be interpreted cautiously, as circulating C1q alone does not directly indicate classical pathway activation (110). Given that marked activation often consumes C1q/C1r/C1s, persistent C1q elevation may instead reflect inflammation-associated upregulation or moderate pathway engagement (111). Contrast agents induce osmotic injury in TECs, leading to mitochondrial dysfunction and DAMP release. Immune complex deposition may further provide a substrate for complement activation (112). Subsequent complement activation may promote MAC deposition on the renal tubular basement membrane, causing cell lysis, ATP depletion, and further DAMP release, thereby forming a positive feedback loop of complement activation that may aggravate renal injury (113, 114). Concurrently, contrast agents promote the release of advanced glycation end products through osmotic injury, disrupting the endothelial glycocalyx and exposing collagen and glycoproteins (115, 116), thereby creating a surface context favorable for MBL/ficolin recognition and MASP-dependent lectin pathway activation (117). MASP-2 mediates C3 activation through the C4bC2b convertase shared by the lectin and classical pathways, promoting inflammatory cell infiltration and potentially contributing to interstitial fibrosis and chronic injury, and amplifying the complement cascade (118). Further complement amplification may lead to C3b deposition, microthrombus formation, and local ischemia, thereby exacerbating renal medullary hypoxia (54). Clinical studies have reported elevated urinary MBL and MASP-2 levels in patients undergoing percutaneous coronary intervention (119), and MBL levels in CI-AKI patients correlate positively with renal injury severity, suggesting that lectin pathway activation may be associated with CI-AKI progression. Ficolin-3 may also participate in complement-mediated renal injury via the MASP-2 pathway (120).

In addition to the classical and lectin pathways, factor H, a key negative regulator of the alternative pathway, may also be involved in CI-AKI. Factor H dysfunction can lead to excessive complement activation, promoting inflammation and apoptosis and accelerating renal injury (121, 122). Moreover, contrast agents can disrupt the phospholipid bilayer of renal tubular cell membranes through osmotic effects, exposing phosphatidylserine as an anchor site for C3b, and releasing DAMPs such as high-mobility group box 1 (HMGB1) (123), thereby activating the alternative pathway through signaling dependent on Toll-like receptor 4 (TLR4)/nuclear factor-κB (NF-κB) signaling (124). Concurrently, HMGB1 released during ischemia can further trigger activation of the classical pathway, thereby exacerbating sterile inflammation and complement amplification reactions (124). Overall, complement activation may contribute to tubular injury, microvascular alterations, and inflammatory amplification in cisplatin- and contrast agent-associated AKI. In CI-AKI, clinical evidence mainly supports an association between complement markers and renal injury severity.

#### 3.2.2 Distinct complement pathways in animal toxin-induced AKI

In animal toxin-induced AKI, complement activation appears to reflect a multi-pathway process rather than activation of a single dominant pathway. The classical pathway is primarily linked to toxin-induced hypersensitivity reactions and immune-inflammatory activation, leading to increased production of C3a and C5a (125). The lectin pathway primarily mediates the recognition of toxin-associated DAMPs. In AKI caused by bee venom or snake venom, toxins rich in phospholipase A2 and hemolytic toxins can directly damage cell membranes and induce renal tubular epithelial cells to release DAMPs (126). These danger signals may be recognized by MBLs, thereby triggering lectin pathway activation (127). Furthermore, toxin-induced secondary hemolysis and rhabdomyolysis can release hemoglobin and myoglobin, and these molecules can further promote the activation of the lectin complement pathway (60). The alternative pathway is characterized primarily by surface-driven amplification. Toxins can directly disrupt the membrane structure of TECs (128) (for example, the bee venom peptide melittin can induce phosphatidylserine exposure (129), exposing negatively charged surfaces that provide a stable platform for C3b deposition and facilitate alternative pathway amplification (130)). Therefore, complement activation in animal toxin-induced AKI is not a single-pathway event, but a continuous multi-pathway pathogenic process involving all three complement activation pathways.

### 3.3 Sepsis-associated AKI

Sepsis-associated AKI (SA-AKI) is one of the most common forms of AKI in critically ill patients. Approximately 50% of patients with sepsis develop AKI within 7 days of onset (131, 132). Unlike ischemic or purely nephrotoxic AKI, a defining feature of SA-AKI is that it cannot be explained solely by hypoperfusion. Under the combined effects of hyperperfusion and a dysregulated immune microenvironment, the kidneys become a highly vulnerable target organ (133). In experimental models, sepsis is typically induced by cecum ligation and perforation (134), and renal oxidative stress, together with activation of the complement and coagulation systems, may contribute to the pathophysiological basis of SA-AKI (135, 136). Mechanistic insights into complement-mediated injury in SA-AKI come mainly from experimental sepsis models, while human studies more often link complement activation to disease severity and outcomes, suggesting a contributory role in SA-AKI progression.

#### 3.3.1 Pathogen-triggered complement initiation and immune dysregulation

In the early stages of SA-AKI, pathogens and their associated molecular patterns can directly initiate the complement cascade and disrupt immune homeostasis (137, 138). The classical pathway is initiated by C1q recognizing IgM or IgG antibodies, while complement activation products such as C3a and C5a promote immune cell recruitment and inflammatory mediator release (139). For example, peptidoglycan and lipoteichoic acid, components of the *Staphylococcus aureus* cell wall, can trigger activation of the classical pathway and lead to the deposition of MAC on the surface of vascular endothelium, thereby aggravating infection-associated vascular injury (140). Concurrently, peptidoglycan can promote red blood cell lysis, and the enhanced deposition of C3b and C4d on the surface of red blood cells in patients with sepsis supports a potential role for complement-mediated hemolysis in worsening SA-AKI (141). Correspondingly, C5b-9 deposition can also be detected in the renal tubulointerstitium (142). Furthermore, pentaxin 3 (PTX3) can bidirectionally regulate the classical pathway by interacting with C1q, and its elevation is strongly associated with poor prognosis in patients with severe sepsis (143, 144). In clinical sepsis, complement fragment deposition and elevated PTX3 levels have been associated with disease severity and worse outcomes, supporting their relevance as markers of immune dysregulation. The lectin pathway is also involved in the early initiation of SA-AKI. MBLs or ficolins recognize microbe-associated molecular patterns, such as bacterial mannose residues and fungal β-glucans, and activate MASP-1, thereby initiating downstream complement activation (145). Concurrently, the combined accumulation of pathogen-associated molecular patterns (PAMPs) and DAMPs can further perturb immune homeostasis and amplify the inflammatory response (131).

#### 3.3.2 Alternative pathway amplification and complement regulatory dysfunction

In experimental SA-AKI models, the alternative pathway appears to be an important amplification pathway and is clinically associated with sepsis-associated disseminated intravascular coagulation and mortality risk (146, 147). Lipopolysaccharide from Gram-negative bacteria can bypass C1q and MBL, directly promoting C3 hydrolysis and triggering alternative pathway activation (139). Its dysregulated amplification is closely linked to an imbalance in complement regulatory proteins: FH and FHRs determine on which surfaces complement activation occurs (33). FH is a major soluble regulator of complement activation that modulates complement activation in both the classical and alternative pathways. Similar to C1q, FH binds to target surfaces through charge-dependent interactions (148), acts at both the C3 and C5 levels, and is primarily synthesized by the liver as a soluble plasma protein (149). Factor H prevents the formation of the terminal C5b-9 complex by interacting with C3b and regulating complement activation on blood and cell surfaces (30, 31). Low FHR2 levels may predispose to complement overactivation (57), whereas 2-O-desulfated heparan oligosaccharides specifically inhibit the binding of FHR1 and FHR5, thereby reducing C3b deposition on the endothelial surface (150). Given its limited intrinsic complement regulatory reserve, the kidney relies heavily on plasma factor H. Consequently, renal dysfunction makes the kidneys susceptible to complement-mediated inflammatory damage (14). Additionally, it can bind to uromodulin and participate in maladaptive repair and the AKI-to-CKD transition (32).

#### 3.3.3 Infection-driven complement–TLR amplification in SA-AKI

In tubular injury during SA-AKI, complement activation appears to interact closely with Toll-like receptor/nuclear factor-κB signaling, forming a feed-forward inflammatory loop. Factor B is a component of the complement alternative pathway. Its expression is upregulated in renal tubular epithelial cells through pattern recognition receptors such as TLR2 and TLR4 (151). PAMP- and DAMP-driven TLR2/TLR4 activation can increase tubular inflammatory signaling and favor alternative pathway amplification (152, 153). This bidirectional interaction supports a sepsis-specific feed-forward inflammatory loop, although the strongest evidence remains preclinical (154, 155).

### 3.4 Intravascular hemolysis- and rhabdomyolysis-associated AKI

AKI is an important complication of intravascular hemolysis (IH) and rhabdomyolysis (RM), which may occur in autoimmune, infectious, genetic, or muscle-injury-related contexts (156–159). Although IH and RM differ in etiology, both release hemoglobin, myoglobin, and heme derivatives, thereby promoting oxidative stress, complement amplification, local inflammation, and tubular injury.

#### 3.4.1 Heme-driven complement initiation and classical pathway activation

In IH/RM-associated AKI, hemoglobin (Hb) released during hemolysis, myoglobin (Mb) released during myolysis, and their heme derivatives can initiate and amplify complement activation. Hb or Mb can form immune complexes with endogenous IgM/IgG and bind C1q, thereby triggering the classical pathway (160). In parallel, externalized cell membrane phospholipids can be recognized by C1q or C-reactive protein, promoting C3b deposition and MAC assembly, which may injure tubular epithelial and endothelial cells (161, 162). Free heme can also bind C1q directly and activate the classical pathway independently of antibodies (163). Subsequent MAC formation may induce cell lysis and the release of hemoglobin and pro-inflammatory mediators, generating a self-amplifying inflammatory loop (164, 165). In addition, Fe²^+^ released from Hb/Mb can enhance C1q binding to IgM/IgG, and iron-bound C5 may be converted into a C5b-like structure in the presence of H_2_O_2_, further aggravating IH-associated AKI (98).

In patients with rhabdomyolysis-associated AKI, massive myoglobin release can promote renal iron deposition and trigger oxidative stress and inflammation through heme–iron reactions (166, 167). Myoglobin-derived heme may also activate the alternative and lectin pathways (60), accompanied by increased levels of complement-related molecules such as C1q, C3, C5a, MBL-A, C5b-9, and CD59 (168).

#### 3.4.2 Lectin and alternative pathway amplification in hemolysis/rhabdomyolysis-associated AKI

In hemolytic settings, heme can directly interact with C3 and factor I, inhibiting factor I-mediated C3b degradation and thereby promoting C3 convertase assembly and complement amplification (169). Clinically, elevated plasma free hemoglobin during extracorporeal membrane oxygenation is closely associated with AKI, likely reflecting hemolysis-related release of hemoglobin, heme, and membrane microvesicles into the circulation (170, 171). The erythrocyte membrane itself may also promote heme-dependent alternative pathway activation (172).

**Figure 3:**
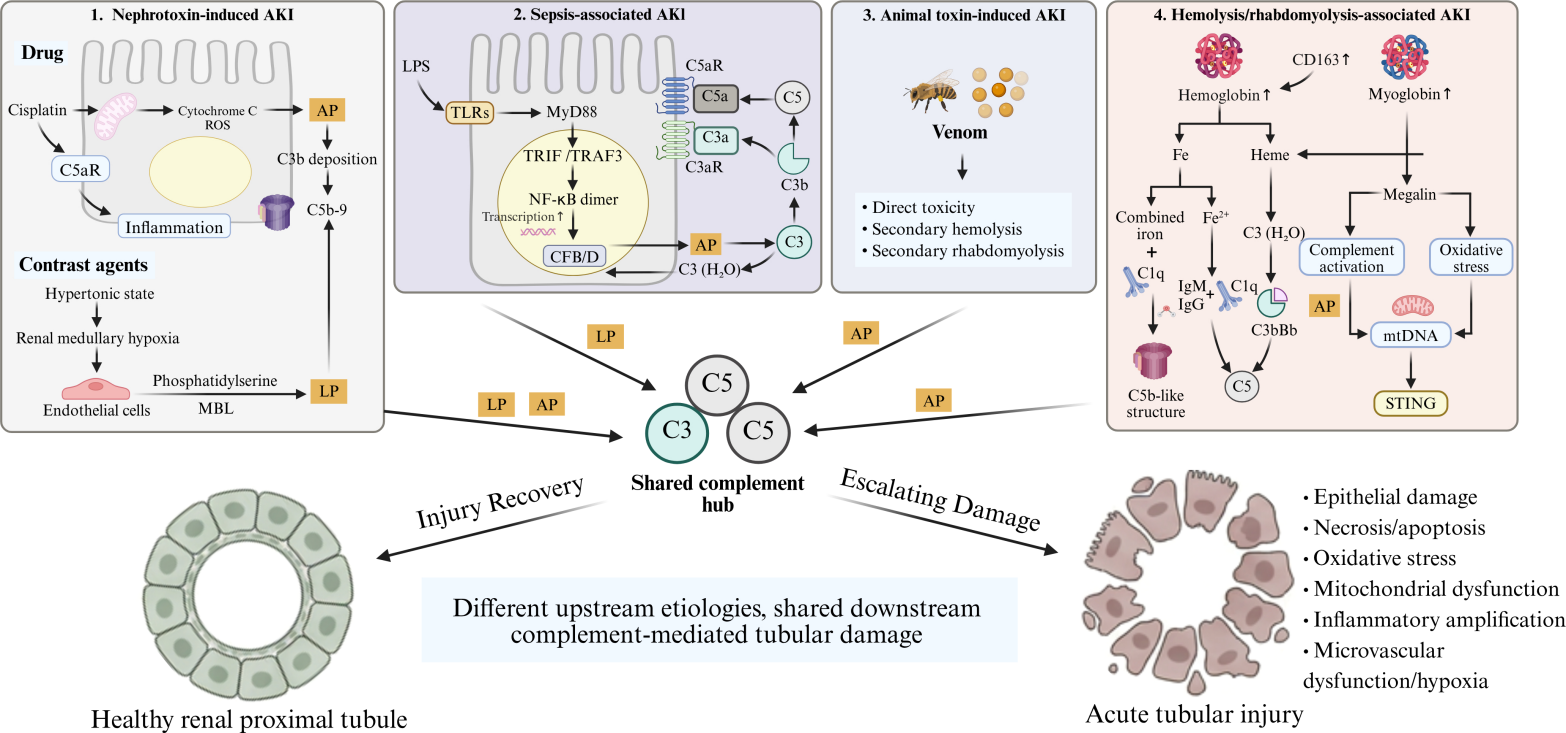
Etiology-specific triggers of complement activation in AKI. Nephrotoxins, sepsis, pigment-related injury, and animal toxins can activate complement through distinct but partially overlapping mechanisms. Cisplatin-induced tubular stress and DAMP release may promote complement activation with possible classical and lectin pathway involvement, whereas contrast agents may facilitate lectin and alternative pathway activation through endothelial glycocalyx disruption, phosphatidylserine exposure, and DAMP release. In sepsis, PAMPs and DAMPs promote complement initiation and alternative pathway amplification through innate immune signaling. In hemolysis- and rhabdomyolysis-associated AKI, hemoglobin, myoglobin, heme, and iron-derived oxidative stress may amplify complement activation and tubular injury. These upstream triggers converge on C3/C5 activation, inflammatory amplification, and MAC-mediated tissue injury. Abbreviations used in the figure: AP, alternative pathway; LP, lectin pathway; TLRs, Toll-like receptors; MyD88, myeloid differentiation primary response 88; TRIF, TIR-domain-containing adaptor-inducing interferon-β; TRAF3, TNF receptor-associated factor 3; CFB/D, complement factor B/factor D; mtDNA, mitochondrial DNA; STING, stimulator of interferon genes. This Figure is created with https://www.BioRender.com.

In rhabdomyolysis-associated AKI, intratubular C3 deposition correlates with AKI severity and is accompanied by enrichment of inflammatory and apoptotic transcriptional programs (173). C3b and iC3b engage CR1 and CR3, respectively, mediating complement effector functions, phagocytosis, and inflammatory amplification (174, 175), while surface-bound C3b further amplifies the alternative pathway and promotes C5a and C5b-9 generation (176). Defects in complement regulators such as CD59 and DAF may further aggravate complement-mediated hemolysis and tissue injury (177, 178). Thus, complement may contribute to IH/RM-associated AKI through surface amplification, receptor-mediated inflammation, and regulatory failure (**Figure 3**).

### 3.5 Virus-associated AKI

#### 3.5.1 SARS-CoV-2-associated AKI

AKI is common among patients with COVID-19 and is associated with increased mortality (179). In SARS-CoV-2-associated AKI, complement activation has been implicated in renal injury and systemic inflammation. Clinically, increased circulating complement activation products or altered complement levels have been reported in severe COVID-19 and are associated with disease severity (61) and adverse outcomes, suggesting that complement dysregulation may reflect or participate in inflammatory amplification during severe disease (62, 63). Within the kidney, complement activation appears to be pathway- and compartment-dependent. MBL and MASP-2 deposition has been observed in the glomeruli, peritubular capillaries, and renal arteries of COVID-19 patients (180), and the extent of complement deposition correlates with renal injury severity (181). SARS-CoV-2 may engage MBL through its spike glycoprotein and activate MASP-2 (182), while virus-induced endothelial injury and DAMP release, including HMGB1, may further enhance local complement activation (183, 184). In addition, postmortem kidney tissue from critically ill patients with COVID-19 showed more pronounced tubulointerstitial C3d and properdin deposition than bacterial sepsis, particularly in peritubular capillaries, suggesting prominent alternative pathway activation (185).

**Figure 4:**
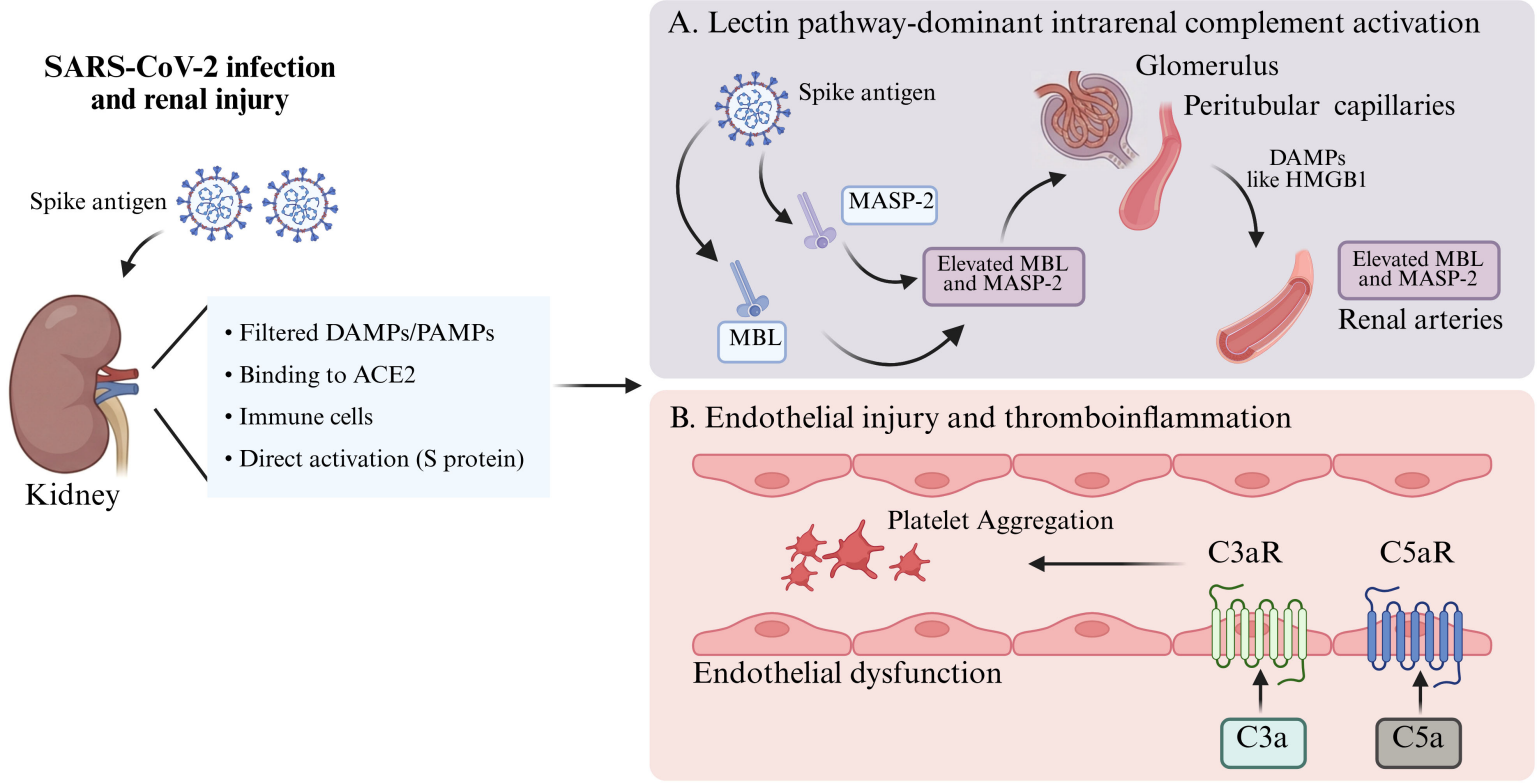
Mechanism of COVID-19-induced complement activation in AKI. During COVID-19, SARS-CoV-2-associated renal injury may be promoted by filtered PAMPs/DAMPs, binding to ACE2, immune cell involvement, and direct effects of the viral spike protein. **(A)** In the kidney, complement activation appears pathway- and compartment-dependent. Lectin pathway activation may involve MBL/MASP-2 deposition in glomeruli, peritubular capillaries, and renal arteries, whereas C3d and properdin deposition in peritubular capillaries suggests alternative pathway amplification. **(B)** Complement effector signaling contributes to endothelial dysfunction and thromboinflammation, as C3a and C5a act through C3aR and C5aR, respectively, and are associated with platelet aggregation and vascular injury. These processes together promote complement-mediated renal damage in COVID-19-associated AKI. Created with https://www.BioRender.com.

Mechanistic studies further suggest that SARS-CoV-2 infection may be accompanied by classical and terminal pathway activation. In a K18-human angiotensin-converting enzyme 2 (K18-hACE2) transgenic mouse infection model, complement components such as C2, C4b, C8 gamma chain, and C9 were significantly upregulated in renal tissue, accompanied by high human ACE2 protein expression, extracellular matrix accumulation, and marked renal dysfunction (186). During infection, serum C3a, C4d, and C5a levels increase, followed by formation of the terminal complement complex C5b-9 (187). Among these mediators, C5a can promote tissue factor expression, thrombomodulin loss, leukocyte recruitment, and a local procoagulant state through C5aR1 (188). The C5a–C5aR axis (189) and MAC–endothelial interactions (190) may jointly contribute to thromboinflammation and organ injury. Overall, complement activation in SARS-CoV-2-associated AKI may contribute to renal and systemic inflammatory injury through lectin pathway activation, C5a/C5aR signaling, NET formation, cytokine storm responses, and thromboinflammation (191). Eculizumab may mitigate terminal complement-mediated pro-inflammatory and prothrombotic effects by inhibiting C5 cleavage. However, its clinical efficacy in AKI remains to be clarified (182) (**Figure 4**).

#### 3.5.2 Other virus-associated AKI

In addition to SARS-CoV-2, influenza viruses, hantaviruses, and HIV may also be associated with AKI, although the role of complement varies across viral contexts. Severe influenza A infection may cause AKI in the setting of critical illness, rhabdomyolysis, disseminated intravascular coagulation, or hemolytic uremic syndrome (192), with complement-mediated injury best supported in influenza-triggered atypical hemolytic uremic syndrome, particularly in patients with alternative pathway dysregulation (193). By contrast, acute Puumala hantavirus-associated nephropathia epidemica shows elevated sC5b-9 and reduced C3 without significant C4 consumption, suggesting alternative pathway activation that correlates with disease severity (194). HIV-associated AKI is multifactorial, and complement involvement is better documented in HIV-associated immune-complex kidney disease, characterized by C3-dominant deposition, hypocomplementemia, and elevated sC5b-9 levels that may improve after antiretroviral therapy (195). Overall, virus-associated AKI is not driven by a single complement mechanism. Complement activation may serve as a severity marker, a contributor to immune-complex-mediated injury, or an amplifier of inflammation.

## 4 Convergent downstream pathogenic programs of complement in AKI

Despite distinct upstream etiologies, complement-dependent injury pathways converge on shared downstream programs, including C3a/C5a signaling, complement–Toll-like receptor crosstalk, NET formation, endothelial-microvascular injury, tubular mitochondrial stress, and maladaptive repair. Rather than acting as an isolated cascade, complement activation integrates with cellular stress and innate immune networks to amplify renal injury (196).

### 4.1 Complement-driven endothelial-microvascular dysfunction and thromboinflammation

Across many etiologic contexts, complement effector molecules such as C5a and MAC can promote endothelial activation and microvascular dysfunction (197). This endothelial stress manifests not only as glycocalyx shedding and upregulation of adhesion molecules, including P-selectin, ICAM-1, VCAM-1, but also further fuels local thromboinflammation (198). Furthermore, MAC deposition induces tissue factor expression and von Willebrand factor release, activating the coagulation cascade and accelerating microthrombi formation (199). Critically, glycocalyx degradation not only compromises barrier function but also impairs the recruitment and anchoring of factor H on the endothelial surface, thereby relieving local constraints on the alternative pathway and converting the injured microvascular surface into a nidus for complement amplification (200). This self-amplifying feed-forward loop ultimately drives progressive deterioration of capillary perfusion, perivascular hypoxia, and failure of microvascular repair.

### 4.2 Sublytic MAC and C5a in mitochondrial stress and programmed cell death

Renal tubular epithelial cells (TECs) represent a major target of injury across diverse forms of AKI. Complement effector molecules can disrupt TEC homeostasis, particularly the assembly of sublytic MACs on the cell membrane. MAC-formed transmembrane pores trigger aberrant Ca2+ influx, thereby precipitating profound mitochondrial dysfunction (201). Hallmarks include sustained opening of the mitochondrial permeability transition pore, depolarization of the mitochondrial membrane potential, excessive ROS generation, and rapid depletion of ATP (202). This complement-driven mitochondrial stress disrupts cellular bioenergetic and survival homeostasis, substantially lowering the threshold for apoptotic and other regulated cell death pathways and pushing TECs beyond reversible stress into irreversible apoptosis, necroptosis, and pyroptosis (203). Concurrently, acute stress further disrupts TEC polarity, resulting in loss of basolaterally localized protective complement regulators, thereby significantly impairing local complement inhibitory capacity (44, 45). Consequently, the surface of damaged tubules becomes more permissive to sustained complement amplification, accompanied by C3 deposition and exacerbated C5b-9-mediated damage, ultimately converting an initially localized metabolic crisis into irreversible parenchymal necrosis (204).

### 4.3 Feed-forward amplification of innate immunity, inflammasome activation, and maladaptive repair

At the downstream effector level, C3 and its activation products can directly reprogram the local immune microenvironment, promoting macrophage polarization toward a classically activated pro-inflammatory phenotype (205). At the same time, the complement system forms a feed-forward amplification loop with Toll-like receptor (TLR) and inflammasome signaling. Complement activation can amplify TLR-driven inflammatory signaling and provide priming signals for the NLRP3 inflammasome (206). This dual-hit axis linking membrane receptors to intracellular sensors may contribute to sustained caspase-1 activation, which mediates robust and sustained release of mature pro-inflammatory cytokines such as IL-1β and IL-18, rapidly transforming initially localized cellular stress into a self-propagating inflammatory cascade (207). Therefore, complement activation may act not only as a consequence of acute injury but also as an amplifier of sustained inflammation, maladaptive repair, and progression to persistent injury.

## 5 Complement-targeted therapeutic opportunities in AKI

AKI, a severe clinical syndrome linked to systemic inflammation and gut microbiota dysbiosis (208), still lacks a disease-specific therapy. However, advancements in hemodialysis technology, complement inhibition therapies, and emerging biomarkers open new avenues for targeted interventions (209). Complement-targeted therapies hold promise for precision medicine by tailoring drug mechanisms to specific renal conditions (210). However, these approaches should not be interpreted as broadly applicable AKI treatments, because most supporting evidence remains preclinical or limited to selected biomarker-defined clinical contexts (**Table 2**).

**Table 2:** Clinical trials of complement-related interventions in AKI.

Target	Drug	Research design	Sample size	Experimental state	Stage	Clinical trial
C1 esterase	Conestat alfa	Randomized, Placebo-controlled, Double-blind, Single-center trial	80	Completed	Phase 2	NCT02869347
		Randomized, Placebo-controlled, Double-blind, Multicenter trial	29	Terminated	Phase 2	NCT04912141
		Randomized, Placebo-controlled, Double-blind, Multicenter trial	250	Recruiting	Phase 2	NCT05145283
C5	Ravulizumab	Randomized, Placebo-controlled, Double-blind, Multicenter trial	32	Unknown status	Phase 3	NCT04570397
		Randomized, Placebo-controlled, Double-blind, Multicenter trial	736	Recruiting	Phase 3	NCT05746559
	Eculizumab	Randomized, Multicenter trial	66	Recruiting	Phase 3	NCT05726916
Serine protein hydrolase	Nafamostat	Randomized, Multicenter trial	160	Unknown status	Phase 3	NCT01486485
		Randomized	60	Completed	Phase 4	NCT02478242
		Randomized, Multicenter trial	66	Completed	Phase 4	NCT01761994
		Randomized, Placebo-controlled, Double-blind, Multicenter trial	166	Recruiting	Not applicable	NCT06150742

From a translational perspective, complement-targeted strategies should be guided by the dominant AKI phenotype, complement biomarker profile, and timing of intervention. Upstream classical/lectin pathway inhibition may be more relevant to immune-complex- or lectin pathway-associated injury, whereas C5 or C5aR blockade may be more suitable for inflammatory or thromboinflammatory phenotypes. Strategies aimed at restoring complement regulation may be particularly relevant when alternative pathway amplification or impaired surface protection predominates (10, 210, 211). Early studies have focused primarily on inhibition of central complement effectors, particularly C3 and C5. In preclinical models, reduction of C3 levels (e.g., *via* C3 siRNA or C3 deficiency) or blockade of upstream complement activation with small interfering RNAs or anti-C1 antibodies improves renal outcomes (212). Inhibition of other complement components, such as C5 (*via* monoclonal antibodies or C5 deficiency) and factor B (monoclonal antibodies against factor B) (182, 213–215), can attenuate MAC formation, tubular injury, neutrophil infiltration, and renal functional decline. In IR models in rodents, elevated expression of the C5a receptor in renal TECs has been observed (216). In rodent IR-AKI models, pharmacologic C5aR antagonism or genetic suppression of C5aR significantly reduced inflammatory mediator levels in renal tissues, alleviated tubular injury without affecting MAC formation (211). Complement components such as C1 and C4b are regulated by decay mechanisms like C1INH, which inactivates C1 esterase and MASP (217). C1q neutralization or depletion of C1q/IgM suppresses classical pathway activation, resulting in near-complete absence of C3 and C4 deposition. Reintroduction of C1q or IgM restores classical pathway activity (218, 219).

Loss or dysfunction of complement regulatory factors may exacerbate AKI progression. For instance, factor H and factor I inactivate C3b and C4b, involving additional factors such as MCP and CR1. C4-binding protein regulates C4bC2b activity by accelerating its decay and serving as a cofactor for factor I-mediated cleavage of C4b (10), thereby limiting C4b-dependent convertase activity. Mice lacking CD59, a membrane-bound complement regulator that prevents C8 binding to C9 and suppresses MAC formation, exhibited enhanced MAC formation during IR injury (220, 221).

Furthermore, the combination of glucocorticoids with immunosuppressive agents, such as anti-CD20 or anti-B-cell activating factor monoclonal antibodies, may attenuate ongoing renal inflammation and tissue injury (213). Membrane-bound complement regulators (e.g., CR1, CD46, CD55, CD59) and soluble factors (e.g., C1INH, factor H, factor I, and S proteins) play central roles in complement regulation. Targeting these regulators may enable selective modulation of distinct stages of the complement cascade. Polymethyl methacrylate-based continuous veno-venous hemofiltration should be considered an extracorporeal supportive strategy that may modulate systemic complement activation, rather than a specific complement inhibitor (210).

In summary, complement-targeted interventions in AKI should be developed as biomarker-guided and phenotype-specific strategies, with careful distinction between experimental efficacy and clinically validated benefit.

## 6 Conclusions and future perspectives

In recent years, the complement system has emerged as an important pathogenic contributor in AKI, although its relative importance varies across etiologies and evidentiary contexts. Across diverse etiologies, including ischemia-reperfusion injury, sepsis, nephrotoxic injury, hemolysis/rhabdomyolysis, and virus-associated AKI, distinct upstream insults converge on C3/C5-centered complement effector programs that promote endothelial-microvascular dysfunction, tubular injury, inflammatory amplification, and maladaptive repair. The extent and nature of complement involvement vary across etiologies, ranging from experimentally supported pathogenic mechanisms to clinically observed associations with disease severity and outcomes. Importantly, complement activation in AKI is not uniform, but exhibits pronounced spatiotemporal heterogeneity, with its pathogenic impact shaped by the renal compartment, injury stage, and local complement regulatory context. These advances not only deepen our understanding of AKI pathogenesis but also underscore the translational potential of complement-related biomarkers and complement-targeted interventions in precision medicine. Although complement inhibitors have shown promising renoprotective effects in experimental and selected clinical settings, broader application requires further validation of patient selection, treatment timing, efficacy, and safety. Future studies should focus on biomarker-guided stratification, stage-specific and compartment-targeted modulation of pathological complement activation, and rational combination strategies that preserve host defense while maximizing renoprotection.

## Glossary

AKI: Acute kidney injury
NET: Neutrophil extracellular trap
C4bC2b: Classical/lectin pathway C3 convertase
MAC: Membrane attack complex
MBL: Mannose-binding lectin
MASPs: Mannose-binding lectin-associated serine proteases
FH: Factor H
FI: Factor I
C1INH: C1 inhibitor
C4BP: C4-binding protein
CKD: Chronic kidney disease
FHRs: Factor H-related proteins
TECs: Tubular epithelial cells
Crry: Complement receptor 1-related protein y
IR: Ischemia-reperfusion
IR-AKI: Ischemia-reperfusion-induced acute kidney injury
CL-11: Collectin-11
THP: Tamm-Horsfall protein
ROS: Reactive oxygen species
CI-AKI: Contrast-induced acute kidney injury
DAMPs: Damage-associated molecular patterns
HMGB1: High-mobility group box 1
TLR4: Toll-like receptor 4
NF-κB: Nuclear factor-κB
SA-AKI: Sepsis-associated acute kidney injury
PTX3: Pentaxin 3
PAMPs: Pathogen-associated molecular patterns
IH: Intravascular hemolysis
RM: Rhabdomyolysis
Hb: Hemoglobin
Mb: Myoglobin
K18-hACE2: K18-human angiotensin-converting enzyme 2
ACE2: Angiotensin-converting enzyme 2
TLR: Toll-like receptor
